# Childhood and adolescent poisoning: a prospective analysis from the largest poison control centre

**DOI:** 10.1186/s12887-026-07062-4

**Published:** 2026-06-09

**Authors:** Salman Muhammad Soomar, Muhammad Awais, Muskaan Abdul Qadir, Anoosha Sohail, Omar Irfan, Abia Abdullah, Mohammad Aadil Qamar, Babar Irfan, Muhammad Omer Sultan, Nadeem Ullah Khan

**Affiliations:** 1 King’s College Hospital London, Dubai, UAE; 2 Medical College, Jinnah Sindh Medical University, Karachi, Pakistan; 3 Aga Khan University, Karachi, Pakistan; 4 Internal Medicine, University of New Mexico School of Medicine, Albuquerque, USA; 5 Independent Consultant, Milton, ON Canada; 6 Medical Unit-1, Jinnah Postgraduate Medical Centre, Karachi, Pakistan; 7 Medical College, Aga Khan University, Karachi, Pakistan; 8 Department of Medicine, Ziauddin University, Karachi, Pakistan; 9 Department of Emergency Medicine, Aga Khan University, Karachi, Pakistan

**Keywords:** Poisoning, Childhood, Adolescent, Pediatric, Organophosphate, Animal Bites

## Abstract

**Background:**

Childhood and adolescent poisoning is a significant health problem globally, especially in low- and middle- income countries. Globally, around a million childhood cases of poisoning were reported in 2019. In Pakistan, unintentional childhood and adolescent poisoning is higher, including organophosphates, snake bites, and animal bites. Therefore, this study aims to identify the most common agents causing poisoning, risk factors, management, and outcomes of childhood and adolescent poisoning incidents.

**Methods:**

This study included childhood and adolescent poisoning cases from March to May 2025 presented at Jinnah Postgraduate Medical Center Karachi, Pakistan. A total of 110 children were included in the study between the ages of 0–18 years. Exposure was poisoning, and there were two outcomes: length of hospital stay (days) and status on follow-up (alive/dead). Acute poisoning was suspected in children who presented a clear history of recent exposure, fast onset of symptoms, and clinical characteristics such as vomiting, altered level of consciousness, convulsions, respiratory distress, aberrant pupil size, or distinctive odor. Organophosphate poisoning was diagnosed based on clear exposure history, identification of substance containers, presence of classical cholinergic toxidrome (vomiting, salivation, lacrimation, pinpoint pupils, fasciculations) and clinical response to atropine. Chi-square and Fisher’s exact test were performed, considering a p-value < 0.05 being significant.

**Results:**

The median age was 17 years with an equal distribution of males and females. Organophosphate poisoning (60.91%) was the most reported poisoning, followed by snake bites (21.82%). Only one (0.91%) case had prior poisoning history. Around 82.00% of poisoning cases occurred at home, and 58.18% of cases occurred due to deliberate intent. Organophosphate poisoning (*p* < 0.001), median respiratory rate of 20 (*p* = 0.034), and heart rate of 100 (*p* = 0.007), were significantly associated with longer hospital stay.

**Conclusion:**

This study highlights organophosphate poisoning as the most common cause, often intentional and occurring at home due to easier access and unsafe storage. This study helps in the identification of patients who are prone to a longer hospital stay than others, providing clinicians with a means to personalize patient care.

**Supplementary Information:**

The online version contains supplementary material available at 10.1186/s12887-026-07062-4.

## Introduction

There were 2.2 million cases of poisoning worldwide in 2019, with 940,000 cases involving individuals under the age of 20 [1]. Recent research after 2021 shows that childhood poisoning remains a significant and, in certain cases, growing concern. A 2024 study from China (*n* = 112) found that case proportions were growing through 2021 [2], while data from another study from China (2019–2023) identified 653 pediatric medication exposures, including an increase in purposeful ingestions among adolescents [3]. In Saudi Arabia, nationwide analysis identified 3,009 cases of chemical poisoning (2019–2021), with 76.4% occurring in children aged 1 to 5 years [4]. These data illustrate the ongoing high loads and changing exposure patterns in the post-pandemic period. In high-income countries, poisoning accounts for about 2.00% of all injury-related deaths, whereas in low-income countries, this figure is as high as 5.00% [5]. Similarly, mortality rates due to self-poisoning are 0.50-1.00% in high-income countries as opposed to 10.0–20.0% in low-income countries. This higher morbidity and mortality burden can largely be attributed to various factors such as socioeconomic patterns, agricultural modernization, increasing availability of new drugs (such as opioids and psychotropics), and easy access to over-the-counter (OTC) medications in low- and middle-income countries (LMIC) [6–8].

Acute poisoning cases can further be classified as intentional or unintentional poisoning. A retrospective study in Malaysia found that unintentional poisoning accounted for 96.70% of cases in children up to the age of five, whereas intentional poisoning made up 76.00% of cases among children aged 13 to 18 [9]. A study from China reported, childhood acute poisoning was prevalent, especially in early childhood (607; 34.6%). The most common causes of poisoning in urban areas were chemicals (59; 10.5%) and drugs (266; 47.2%). In rural regions, the most common causes were pesticides (620; 52.1%). Ingestion was typically the route of poisoning (1671; 95.2%); most poisonings were unintentional (1618; 92.3%) [10].

Soave, Paolo Maurizio, et al. (2022) reported from a study conducted in Italy that 51.1% of the population was male, and the mean age was 30 months. Most poisoning cases (90.1%) were unintentional, and the most common cause of poisoning was drug ingestion (39.4%). In 83.7% of instances, acute poisoning occurred at home. There were no recorded deaths [11].

Khan et al. report in their study based on data from the Pakistan National Emergency Department Surveillance (Pak-NEDS) that the percentage of intentional (49.60%) and unintentional (50.40%) attempts was equal, with patients ≤ 24 years old, accounting for 47.70% of the total unintentional population [12]. Common causes of self-inflicted cases of intentional poisoning include failed academic pursuits, unemployment, financial stressors, and interpersonal relationship issues, whereas poisoning inflicted on others may stem from revenge, anger, or hatred [12]. Both intentional and unintentional poisoning pose significant public health concerns for the pediatric population, especially in Pakistan.

According to the Pakistan Bureau of Statistics (PBS), around 40.00% of the population is aged under 15 years as per the 2023 census [13]. In Pakistan, where a large proportion of the population comprises children and where many households lack adequate safety measures, the risk of pediatric poisoning is particularly high. Therefore, this study aims to identify the most common agents causing poisoning, risk factors of poisoning, management, and outcomes of poisoning incidents in children aged 0–18 years.

## Methods

### Study design

This study employed a prospective observational design. Children presenting with poisoning were enrolled and followed from hospital admission through discharge and up to 30 days post-discharge. The exposure of interest was child poisoning, and the primary outcomes were length of hospital stay (in days) and clinical status at follow-up (alive or deceased). A 30-day follow-up assessment was conducted to ascertain survival status. No comparison group was included, as the study aimed to describe clinical course and outcomes within the exposed cohort.

The Strengthening the Reporting of Observational Studies in Epidemiology (STROBE) checklist was used to ensure the comprehensibility and completeness of this study.

### Study setting

This study was conducted at the National Poisoning Control Centre (NPCC), Medical Unit 1 at Jinnah Postgraduate Medical Centre (JPMC), Karachi, Pakistan. NPCC is one of the largest registered position control centers in Karachi, Pakistan, where an inordinate number of poisoning cases are reported each year.

### Participants and eligibility criteria

Children aged 0–18 years admitted to the NPCC during March to May 2025 with a valid medico-legal number and history and clinical features suggestive of acute poisoning were recruited in our study (Supplementary Fig. 1).

Acute poisoning was suspected in children who presented a clear history of recent exposure, fast onset of symptoms, and clinical characteristics such as vomiting, altered level of consciousness, convulsions, respiratory distress, aberrant pupil size, or distinctive odor. A complete exposure history was used to confirm the type of poisoning, which included inspecting the substance container where one was available and evaluating clinical indications compatible with established toxidromes. When necessary, laboratory studies were conducted, such as toxicological screening or the detection of certain biochemical markers. In circumstances where test confirmation was not attainable, the diagnosis was made using a combination of exposure history and clinical presentation.

### Sampling strategy and sample size

The purposive sampling method was used to recruit study participants. All patients presented with poisoning during the study period were included, as no formal line listing was available. The sample size calculation was initially based on detecting a difference in outcomes between two groups in a cohort study, using a formula for two independent proportions. With $\:{p}_{1}=0.25$and $\:{p}_{2}=0.50$, the calculated total sample size was 116, adjusted to 129 to account for a 10% non-response rate.

- Per-group sample size: $\:n=3.590/0.0625=57.44\approx\:58$
- Calculated sample size: $\:n=116$
- Sample size after 10% non-respondent rate: $\:n=129$

Sample size achieved 110 (85.27% power achieved).

However, as the primary aim of this cohort study is descriptive, the final sample size is also justified based on feasibility, considering the expected number of eligible participants available during the study period from hospital records.

### Data collection and management

Data were collected using a structured case report form specifically developed for this study. The form captured detailed demographic and clinical information relevant to acute poisoning presentations. The variables assessed included: history of previous poisoning (yes/no), type of poisoning (organophosphate (OP), sleeping/over-the-counter pills, other chemical agents), animal-related poisoning (snake bite, other animal bites, insect bites), route of exposure (ingestion or skin contact), location of poisoning (home or outside), and intent (intentional or unintentional).

Clinical presentation was documented through a predefined checklist of symptoms including vomiting, diarrhea, lacrimation, pinpoint pupils, fasciculations, swelling, salivation, abdominal pain, and other reported symptoms. Physiological parameters were recorded on arrival, including respiratory rate, heart rate, systolic/diastolic blood pressure, Glasgow Coma Scale (GCS), and oxygen saturation (SpO₂), each summarized as median with interquartile range (IQR). The time interval between exposure and emergency department (ED) presentation was recorded in hours.

Management details captured included administration of atropine, gastric lavage, intravenous normal saline, intravenous Ringer lactate, and intravenous Augmentin. Patient disposition (discharged or admitted), and status (alive or expired) were also documented. Outcome was length of hospital stay (days). Poisoning was diagnosed based on patient history and clinical symptoms, as laboratory confirmation (e.g., blood cholinesterase or toxicology tests) were not available. Information was taken from the patient’s file, and any missing data was obtained from the patient or their family members. Informed written consent was obtained from the patient’s family or guardian. All patients admitted to NPCC undergo a detailed examination by a medico-legal officer and are given specific registration numbers. All patients were referred according to their assigned registration numbers to ensure confidentiality and anonymity while avoiding any biases. The medical-legal formalities were completed as per the institution’s protocol. The collected data were cleaned and transferred to statistical software for analysis.

OP poisoning was diagnosed based on clear exposure history, identification of substance containers, presence of classical cholinergic toxidrome (vomiting, salivation, lacrimation, pinpoint pupils, fasciculations) and clinical response to atropine. Laboratory confirmation such as serum cholinesterase levels was not routinely available. Regarding specific OP compounds, detailed product-level documentation such as chlorpyrifos, malathion, and diazinon was not consistently available in medical records.

### Variables

Exposure was poisoning, and there were two outcomes: length of hospital stay (days) and status on follow-up (alive/dead). Demographic variables included age, gender, marital status, occupation, and area of residence. Variables related to poisoning (type/source of poison, route of ingestion, intent, presenting symptoms, vital signs, and time to hospital presentation) and patient outcomes (treatment received and patient disposition) were extracted.

### Statistical analysis

Qualitative categorical variables were expressed as frequencies and percentages. Quantitative continuous data were expressed as median and intra-quartile range (IQR). A one-way ANOVA test was applied to see the association between age (in years) and intent of poisoning. Data was further stratified on the length of hospital stay (< 4 and ≥ 4 days). A 4-day cut-off for hospital stay because the majority of pediatric poisoning cases are discharged within three days, making ≥ 4 days a practical threshold to identify prolonged admissions (9). Post-stratification Chi-square test for parametric data and Fisher’s exact test for non-parametric data were applied. Multivariable logistic regression analysis was performed reported risk ratio (RR) with 95% confidence interval, considering the p-value < 0.05 as significant. Stata version 16.0 was used for data analysis.

## Results

Of the 110 patients, the male and female distribution was 1:1 in ratio. The median (IQR) age was 17 (9–18) years. Half of the patients were students (*n* = 56, 50.91%).

Most patients resided in urban areas (87.27%, *n* = 96), indicating a higher occurrence of poisoning in urban settings compared to rural areas (12.73%, *n* = 14). The majority had no pre-existing medical conditions (93.64%, *n* = 103) and were not on any regular medications (99.09%, *n* = 109), suggesting poisoning incidents were largely unrelated to chronic illness or prescribed drug exposure. Regarding employment and education status, 50.91% (*n* = 56) were students, while 24.55% (*n* = 27) were unemployed and not studying, and another 24.55% (*n* = 27) were employed and not studying, highlighting adolescents and economically inactive groups as potentially vulnerable populations. In terms of housing conditions, 61.82% (*n* = 68) lived in kutcha houses, compared to 38.18% (*n* = 42) in cemented/brick houses, suggesting lower socioeconomic housing conditions may be associated with increased poisoning risk. (Table 1).

**Table 1:** Socio-demographic characteristics of childhood and adolescent poisoning patients (*n* = 110)

Characteristics	*n* (%)
Age	
Years (median, IQR)	17 (9–18)
Gender	
Male	56 (50.91)
Female	54 (49.09)
Marital status	
Single	98 (89.09)
Married	12 (10.91)
Residence area	
Urban	96 (87.27)
Rural	14 (12.73)
Current Medical Conditions	
None	103 (93.64)
Current Medications	
None	109 (99.09)
Employment Status	
Employed and not studying	27 (24.55)
Unemployed and not studying	27 (24.55)
Student	56 (50.91)
Type of housing	
Kutcha house	68 (61.82)
Cemented/brick house	42 (38.18)
Current Living Situation	
Parent	99 (90.00)
Siblings	1 (0.91)
Spouse/ In-laws	10 (9.09)

*IQR *Inter Quartile Range

**Fig. 1:**
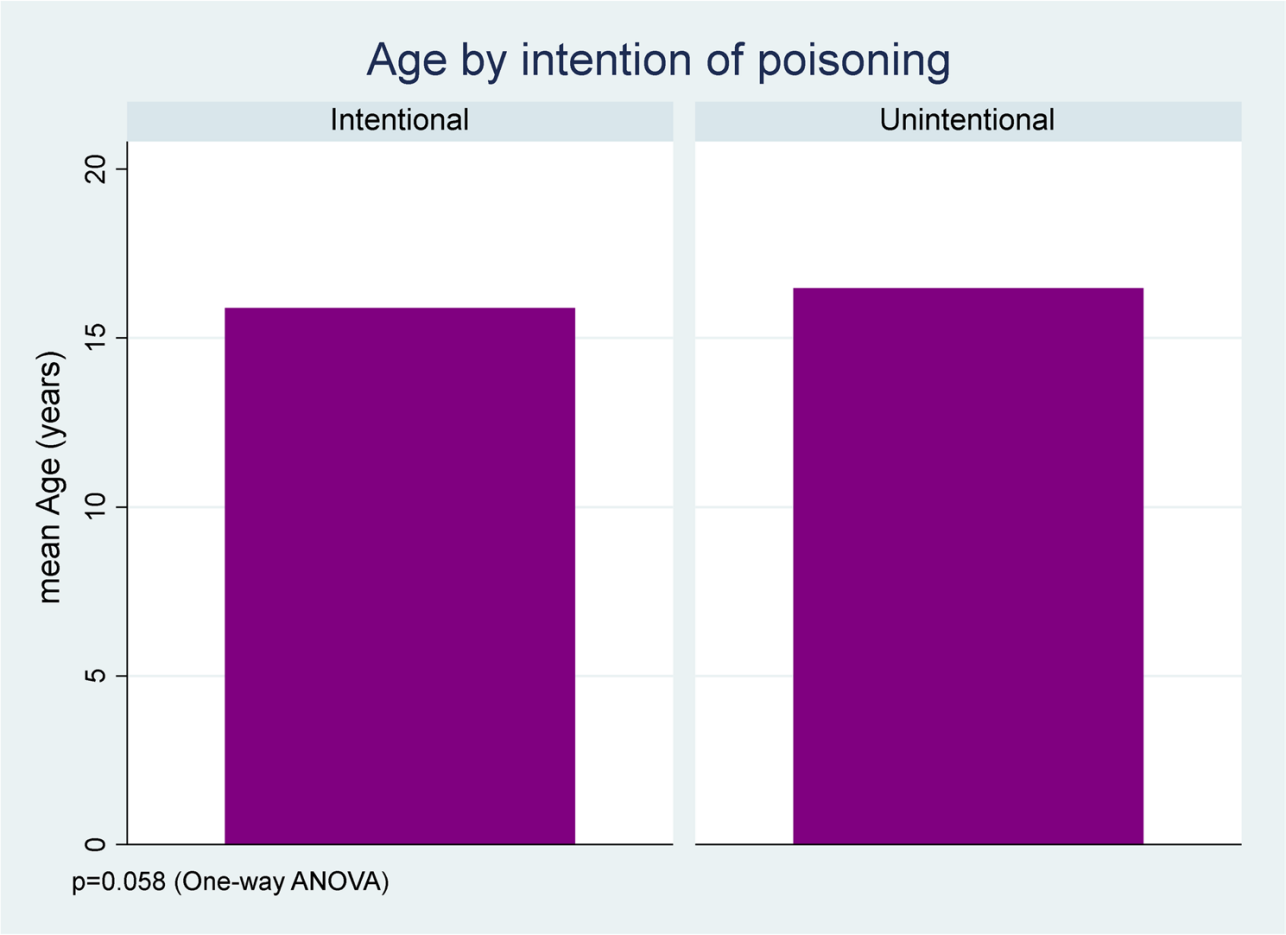
Childhood and adolescent poisoning intent by age (in years)

Mean age was similar in both groups of intentional and unintentional poisoning and age was not significantly associated with intent of poisoning (*p* = 0.058) (Fig. 1).

**Fig. 2:**
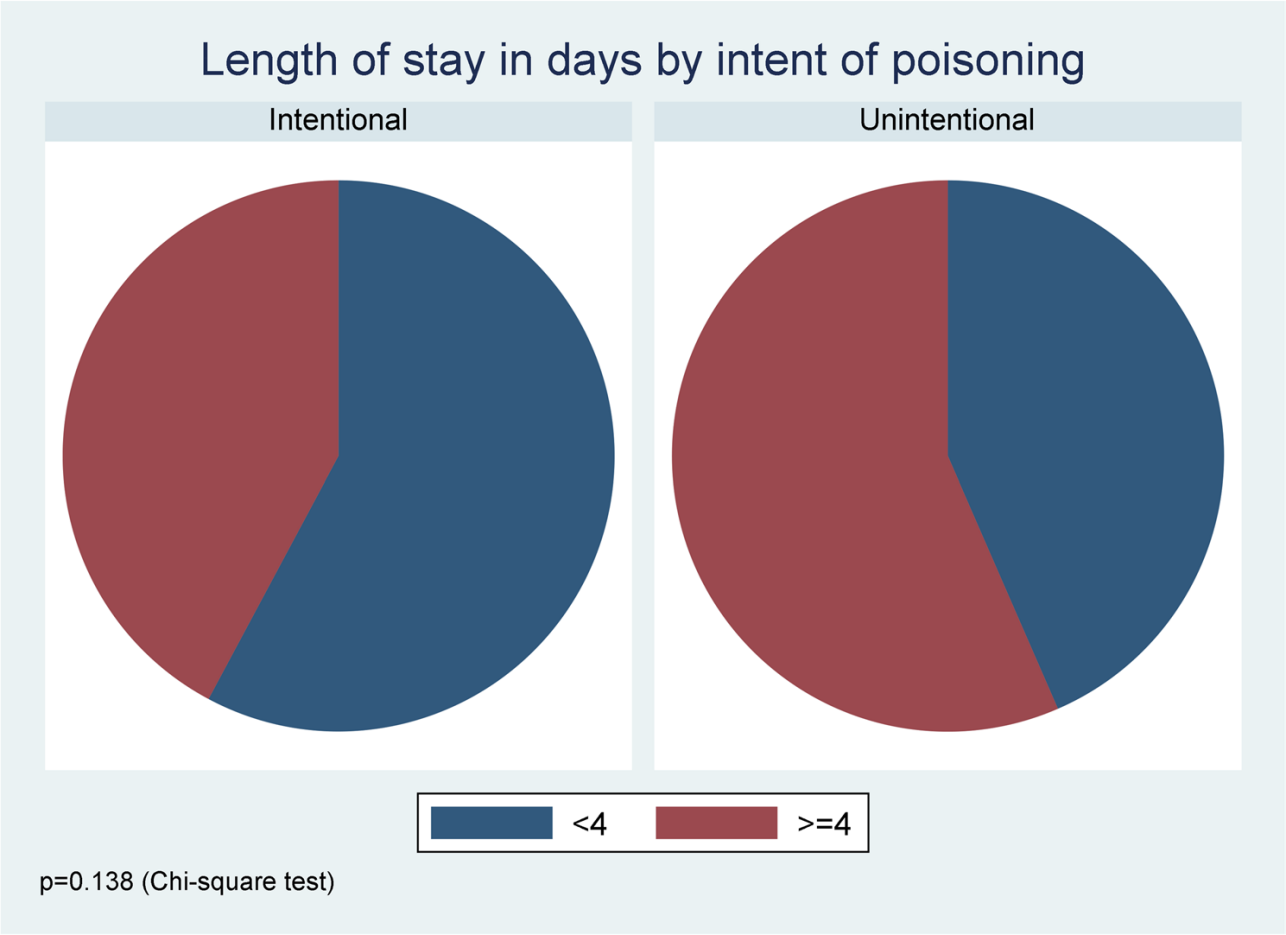
Childhood and adolescent poisoning intent by length of stay (in days)

Of the 110 patients, only 1 (0.91%) had a previous history of poisoning. Most patients had organophosphate poisoning (*n* = 67, 60.91%). Among those 67, 66 (98.51%) children between 13 and 18 years of age had OP poisoning. While the most common route of poisoning was through ingestion (*n* = 83, 75.45%). Most patients ingested poison at home (*n* = 91, 82.73%). The Intent of poisoning was almost 1:1, intentional poisoning was reported by 58.18% patients (*n* = 64) and 41.82% (*n* = 46) unintentional poisoning. Intent of poisoning was medico-legal categorization. Multiple signs and symptoms were reported by the presenting cases, with the most common vomiting (*n* = 86, 78.18%). The time taken from poisoning to presentation to the Emergency Department (ED) was a median of 2 h. The median length of the hospital stay was 4 days (IQR 3–5). No mortality occurred due to poisoning. Snakebite cases (21.82%) were predominantly unintentional and occurred in peri-urban settings. Most required anti-venom administration and supportive management. None resulted in mortality, and the median hospital stay was shorter compared to organophosphate cases. (Table 2) (Fig. 2).

**Table 2:** Clinical characteristics childhood and adolescent patients (*n* = 110)

Characteristics	*n* (%)
Previous History of Poisoning	
Yes	1 (0.91)
No	109 (99.09)
Type of poisoning	
Organophosphate	67 (60.91)
Sleeping/over-the-counter pills	4 (3.64)
Others	13 (11.82)
Type of poisoning (animal related)	
Snake bite	24 (21.82)
Animal bite	1 (0.91)
Insect bite	1 (0.91)
Route of Poisoning	
Ingestion	83 (75.45)
Skin contact	27 (24.55)
Location of poisoning	
Home	91 (82.73)
Outside	19 (17.27)
Intent	
Intentional	64 (58.18)
Unintentional	46 (41.82)
Symptoms	
Vomiting	86 (78.18)
Diarrhea	42 (38.18)
Lacrimation	43 (39.09)
Pinpoint Pupils	49 (44.55)
Fasciculation	53 (48.18)
Swelling	12 (10.91)
Salivation	22 (20.00)
Abdominal Pain	8 (7.27)
Others	13 (11.82)
Respiratory rate	
Breaths per minute (median, IQR)	19 (18–20)
Heart rate	
Beats per minute (median, IQR)	85 (78–99)
SBP/DBP	
mmHg (median, IQR)	70/110 (60/100 − 80/120)
GCS (median, IQR)	15 (13–15)
SPO2	
% (median, IQR)	98 (93.5–99)
Time difference between poisoning and presenting to the ED	
Hours (median, IQR)	2 (1.5-4)
Treatment administered	
Atropine	82 (74.55)
Gastric lavage	25 (22.73)
IV Normal Saline	75 (68.18)
IV Ringer Lactate	72 (65.45)
Inj. Augmentin	84 (76.36)
Disposition	
Discharged	110 (100.00)
Length of stay in hospital	
Days (median, IQR)	4 (3–5)
Outcome	
Alive	110 (100.00)
Expired	0 (0.00)

*IQR *Inter Quartile Range, *SBP *Systolic Blood Pressure, *DBP *Diastolic Blood Pressure, *GCS *Glasgow Coma Scale, *SpO2 * Oxygen Saturation, *IV *Intravenous, *Inj. *Injection

The majority of the organophosphate poisoning patients stayed longer than 4 days in the hospital (*n* = 44, 83.02%), *p* < 0.001. Patients with lower GCS (IQR 10–15) and oxygen saturation (IQR 80–98) had a prolonged stay in hospital (*p* < 0.001). Patients who arrived in the ED with raised respiratory rate (median, IQR 20 (18–24) *p* = 0.034) and heart rate (median, IQR 100 (85–127) *p* = 0.007) stayed longer than 4 days in the hospital. No difference in intentional and unintentional poisoning patients’ length of stay (*p* = 0.138) (Table 3).

**Table 3:** Difference in clinical characteristics with length of hospital stay in childhood and adolescent poisoning patients (*n*=110)

Characteristics	Length of hospital stay (days)		*P*-value
	< 4 days (*n* = 57)	≥ 4 days (*n* = 53)	
Poisoning type			
Organophosphate	23 (40.35)	44 (83.02)	
Sleeping/over-the-counter pills	4 (7.01)	0 (0.00)	< 0.001*ǂ
Other	11 (19.30)	2 (3.77)	
Type of poisoning (animal related)			
Snake bites	19 (33.33)	5 (9.43)	
Animal/ insect bites	0 (0.00)	1 (1.89)	0.573*
Insect bites	1 (1.75)	0 (0.00)	
Respiratory rate			
Breaths per minute (median, IQR)	18 (16–20)	20 (18–24)	0.034**ǂ
Heart rate			
Beats per minute (median, IQR)	85 (78–98)	100 (85–127)	0.007**ǂ
SBP/DBP			
mmHg (median, IQR)	70/110(70/100 − 80/120)	70/110(70/100 − 80/120)	0.618**
GCS (median, IQR)	15 (13–15)	15 (10–15)	< 0.001**ǂ
SPO2			
% (median, IQR)	98 (97–99)	97 (80–98)	< 0.001**ǂ

*Fisher’s exact **ANOVA ǂstatistically significant

Age, intent and type of poisoning were significant predictors of length of stay. The adjusted risk ratio of increased length of stay among patients aged 13–18 years was 1.96 times higher compared to 0–5 years (95% CI: 1.27–3.33, *p* = 0.008). Similarly, the adjusted risk ratio of increased length of stay among intentionally poisoning patients was 1.76 times higher (95% CI: 1.25–2.99, *p* = 0.001) compared to unintentional poisoning patients. Moreover, the adjusted risk ratio of increased length of stay among patients with Organophosphate poisoning was 1.98 times higher (95% CI: 1.37–2.86 *p* < 0.001) compared to other types of poisoning (Table 4).

**Table 4:** Crude (cRR) and Adjusted Risk Ratio (aRR) with 95% CI

Characteristics	cRR (95% CI)	*p*-value	aRR (95% CI)	*p*-value
Age (years)				
0–5	1			
6–12	1.34 (1.17–2.99)	0.009	1.71 (1.18-2.00)	0.008*
13–18	1.65 (1.28–3.58)		1.96 (1.27–3.33)	
Gender				
Female	1			
Male	1.47 (0.86–2.45)	0.443	-	-
Intent				
Unintentional	1		1	
Intentional	1.78 (1.19–3.83)	0.014	1.76 (1.25–2.99)	0.001*
Type of poisoning				
Sleeping/over-the-counter pills	1			
Organophosphate	2.40 (1.79–4.26)	0.020	1.98 (1.37–2.86)	< 0.001*
Others	2.19 (1.73–4.10)		1.47 (1.22–1.90)	
Respiratory rate Breaths per minute (median, IQR)	1.36 (1.17–2.05)	0.031	1.59 (0.94–2.92)	0.500
Heart rate Beats per minute (median, IQR)	0.99 (0.34–1.84)	0.768	-	-
GCS (median, IQR)	1.07 (0.72–2.31)	0.381	-	-
SPO2% (median, IQR)	0.94 (0.60–1.56)	0.746	-	-
Time difference between poisoning and presenting to the ED. Hours (median, IQR)	0.95 (0.63–1.45)	0.459	-	-

*Significant at multivariable analysis

## Discussion

The findings of our study showed that the median age of children who were presented was 17 years, with equal gender distribution. Most patients did not have any documented history of previous poisoning. Most of the patients were students and lived at home with their parents. Organophosphate poisoning was the most common source of poisoning, with many cases occurring at home and with deliberate intent.

In a study from India reported among the age group of 12 to 19 (30.2%). Organophosphorus compound was the cause of most poisoning cases (36.0%) [14]. Organophosphate poisoning was found to be associated with a longer duration of hospital stay (≥ 4 days), findings consistent with previously published studies [15, 16]. Despite this, a continuous infusion after the initial bolus dosage requires several days, while patients may also undergo respiratory failure requiring ventilatory support, which prolongs the hospital stay in patients [17]. Moreover, lower GCS score and SPO2, and increased respiratory and heart rates, were attributed to a prolonged hospital stay, findings consistent with published studies [18–20].

Our study demographic distribution aligns with a previous study investigating acute poisoning cases at NPCC over a five-year period from 2017 to 2021, which also found that most cases occurred in adolescent, with equal gender distribution [17]. In contrast, many single-center studies on acute poisoning in Pakistan have shown varying gender trends, likely due to small sample sizes, differences in poisoning agents, or socioeconomic factors affecting the populations studied [21–24]. However, age patterns have remained consistent throughout studies, with adolescent and teenagers being the most affected groups in cases of intentional poisoning [12, 21, 22].

Moreover, organophosphate poisoning was the most common agent in our study, a finding consistently reported across Pakistan, particularly in cases of intentional poisoning in both rural and urban settings [21]. A scoping review on the determinants and methods of self-harm and suicide in Pakistan highlighted the high prevalence of organophosphate use in both deliberate self-harm and suicide, largely due to its widespread availability and lack of regulations [25]. Similarly, another systematic literature review on suicide by pesticide poisoning in Pakistan found that 47.00% of the poisoning cases in Pakistan were due to pesticides, with organophosphates being the most commonly reported pesticide [25]. Additionally, another cross-sectional study conducted in Lahore reported that 58.00% of Childhood and adolescent poisoning cases involved pesticides, with a mortality rate of 6.20% [12]. Varying rates of mortality have been reported across the literature depending on the dose ingested, type of organophosphate product used, and time taken to reach the ED [12].

While global regulatory frameworks, such as the World Health Organization (WHO), have recommended measures to regulate the use of hazardous pesticides, including organophosphates [26], these chemicals remain widely used in many LMIC, particularly those in South Asia. In countries like Pakistan, where agriculture forms the backbone of the economy, accessibility and affordability contribute to their continued widespread use among farmers. Moreover, the lack of strict regulations on organophosphates allows them to be easily sold in markets including in urban areas as found by a retrospective cohort study at NPCC that focused on demographics and outcomes in organophosphate poisoning patients during 2019 found that of the 3300 cases identified, majority patients were from Karachi and the most common form of organophosphate ingestion were off label products followed by rat killers and drain cleaners [22]. According to WHO estimates, Pakistan accounts for nearly 20.00% of all pesticide-related poisoning deaths in South Asia, with a higher burden in rural areas due to increased availability of organophosphates [27].

Furthermore, our study found that many of the poisoning cases occur at home and are intentional, i.e., more cases of poisoning, i.e., 96 (87.27%), in urban areas. Since the majority of the patients were from Karachi. Also, on intent, the numbers are 64 (58.18%) intentional & 46 (41.82%) unintentional. Since most of the participants in our study lived with their parents, this finding is particularly interesting since parental support is a well-established protective factor against self-harm and suicidal behaviors. However, a study from Pakistan assessing the knowledge, attitudes, and perceptions of parents of children aged 0–16 years found that while 80–97.00% of the parents’ knowledge regarding the harmful effects of poisons and household chemicals was adequate, 98.00% of the parents were unaware of any services to provide first aid in the event of poisoning, thus highlighting the general lack of awareness regarding first aid and poison control centers among caregivers which further contributes to the high morbidity and mortality rates of Childhood and adolescent poisoning in the country [28]. Additionally, a Karachi-based study on unintentional pediatric poisoning reported that 45.00% of cases were due to household chemicals while 30.00% involved pharmaceuticals, primarily analgesics and sedatives. This highlights the role of inadequate safety measures in Pakistani households [28]. Moreover, previous studies on acute poisoning in Pakistan have demonstrated a positive correlation between age and intent of poisoning [21]. Studies focusing on toddlers have reported accidental poisoning as the most common cause (20), whereas a higher prevalence (60.00%) of intentional poisoning has been reported in adolescent and teenagers [22].

The present study on acute poisoning in pediatric patients reveals findings consistent with epidemiological patterns reported in recent literature, particularly regarding organophosphate poisoning. The predominance of OP poisoning (60.91%) among these cases aligns with global data underscoring organophosphates as a major cause of acute poisonings, especially in agricultural and developing regions. The almost exclusive occurrence of OP poisoning in adolescents aged 13–18 years (98.51%) further supports literature demonstrating a higher incidence of pesticide poisoning among adolescents, often attributed to intentional self-harm in this age group [29].

The predominance of ingestion as the route of poisoning (75.45%), predominantly at home (82.73%), corresponds to recognized patterns where oral ingestion remains the most common exposure route in household settings, particularly intentional ingestion for suicidal behavior or accidental ingestion due to unsafe storage. The near equal distribution between intentional (58.18%) and unintentional (41.82%) poisoning underscores the dual nature of pediatric poisoning risks; while unintentional poisoning is common in younger children, adolescents frequently present with intentional poisoning as a form of self-harm [30].

Clinically, the predominance of vomiting as a symptom (78.18%) is consistent with cholinergic effects of OP toxicity, which typically manifest as diverse signs including gastrointestinal disturbances. The median interval of 2 h from exposure to emergency department presentation is relatively prompt compared to reports noting variable admission times that can affect prognosis. Early hospital presentations are critical for effective management, including atropine and oxime therapy, which have been shown to improve outcomes in OP poisoning cases [31].

Although no cases of aluminum phosphide or paraquat poisoning were identified in this cohort, these agents are well-documented contributors to pesticide-related morbidity and mortality in Pakistan. Aluminum phosphide has been associated with high case fatality rates due to the absence of a specific antidote, while paraquat poisoning is similarly linked to severe toxicity and poor clinical outcomes. The absence of such cases in our study may reflect regional variations in pesticide availability, reporting patterns, or sample characteristics. Nonetheless, their established public health burden underscores the need for continued surveillance and regulatory control.

### Strengths and limitations

#### Strengths

This study includes both intentional and unintentional poisoning, providing a more comprehensive perspective than much of the existing literature. The use of validated, standardized reporting guidelines enhances the clarity and transparency of reporting and outcomes. Furthermore, risk factors identified in this study—including lower Glasgow Coma Scale (GCS), reduced oxygen saturation, and organophosphate poisoning were associated with prolonged hospital stay, enabling improved patient stratification and more efficient allocation of limited healthcare resources.

### Limitations

However, several limitations should be acknowledged. First, as a single-center study with a small sample size limited to a pediatric population, generalizability is restricted. Second, selection bias toward older children and adolescents may have influenced the observed proportion of intentional poisoning. In addition, intentional poisoning is considered a medico-legal case in Pakistan, potentially discouraging care-seeking due to legal and social concerns, thereby contributing to underreporting and possible information and selection bias. Third, data on the quantity of poison exposure and the specific type of organophosphate pesticide were unavailable. The absence of detailed case-specific information for certain poisoning types of further limits interpretation. Activated charcoal was not administered due to unavailability at the study center. Standardized severity scores, such as POP (Pediatric Organophosphate Poisoning) and APACHE, were not recorded, limiting objective assessment of poisoning severity. Finally, the study population predominantly comprised older adolescents (> 80% aged ≥ 13 years), precluding meaningful stratification into younger age groups (0–5 and 6–12 years) and limiting applicability to younger pediatric populations. The study does not explore root causes, family backgrounds, or access pathways to toxins. Furthermore, specific OP compounds of product-level documentation (e.g., chlorpyrifos, malathion, diazinon) was not available in medical records. Also, during the study period, no confirmed cases of aluminum phosphide or paraquat poisoning were recorded. Furthermore, laboratory confirmation such as serum cholinesterase levels was not routinely available. Regarding specific OP compounds, detailed product-level documentation such as chlorpyrifos, malathion, and diazinon was not consistently available in medical records. A key limitation of this study is the lack of assessment of important psychosocial factors that may influence the outcomes of interest. Variables such as family conflict, academic stress, and prior mental health history were not collected.

### Recommendations and future directions

Based on our findings, early recognition of cholinergic features and timely administration of atropine should remain a priority in suspected organophosphate exposures, particularly given the delays in presentation observed. Strengthening triage protocols, educating caregivers on early symptom identification, and ensuring availability of antidotes and basic supportive care at peripheral facilities can further improve outcomes. Future research should incorporate laboratory confirmation, explore determinants of delayed presentation, and evaluate the effectiveness of standardized management pathways. Larger multicenter studies with comprehensive toxicology testing are needed to better characterize emerging toxins and refine evidence-based treatment strategies in childhood and adolescent poisoning.

## Conclusion

This study demonstrates that organophosphates were the most common cause of acute poisoning, with most incidents occurring at home, likely due to unsafe storage and unregulated availability. Easy accessibility further increases the risk of both intentional and unintentional poisoning among children and adolescents. By identifying risk factors associated with prolonged hospital stay, the study provides clinically relevant insights for early risk stratification and outcome prediction, enabling more efficient resource allocation. These findings underscore the need for improved storage practices, stronger regulatory oversight, and targeted public awareness initiatives to reduce domestic access to hazardous chemicals.

## Supplementary Information

Supplementary Material 1: Supplementary figure 1: Enrollment flow diagram.

## Data Availability

Data and materials are available from the corresponding author on a reasonable request.

## Abbreviations

JPMC: Jinnah Postgraduate Medical Centre (JPMC)
LMICs: Low-and Middle-Income Countries
NPCC: National Poisoning Control Centre
OTC: Over the Counter
PakNEDS: Pakistan National Emergency Department Surveillance
PBS: Pakistan Bureau of Statistics
STROBE: Strengthening the Reporting of Observational Studies in Epidemiology

## References

[1] Mottla ME, Bowler M-E, Asgary R. Epidemiology, risk factors, and strategies to prevent and manage poisonings due to pharmaceuticals in children in low income and low-middle income countries: A systematic review. J global health. 2023;13:04173. 10.7189/jogh.13.04173 PMC10754493 38154015

[2] Zhang H, Huo Q, Jing R, Dong M. Clinical analysis of acute poisoning in children. BMC Pediatr. 2024;24(1):212. 38528509 10.1186/s12887-024-04697-z PMC10962155

[3] Duan ZY, Qu YN, Tang R, Liu JT, Wang H, Sheng MY, et al. Evaluating the Characteristics and Outcomes of Acute Pharmaceutical Exposure in Children: 5-Year Retrospective Study. JMIR Pediatr Parent. 2025;8:e66951. 40526889 10.2196/66951 PMC12187026

[4] Alshahrani MM, Albogami HA, Asiri AA, Al Haydhah KS, Aldeailej IM, Aldehaim MA, Lubbad MY, Alalyan LA, Alasmari AF, Al Salem IY, Alqahtani A. Epidemiological trends of acute chemical poisoning among children over a recent three-year period in Saudi Arabia. Children. 2023;10(2):295. 10.3390/children10020295 PMC9955334 36832424

[5] Edelu B, Odetunde O, Eke C, Uwaezuoke N, Oguonu T. Accidental Childhood Poisoning in Enugu, South–East, Nigeria. Annals Med health Sci Res. 2016;6(3):168–71. 10.4103/2141-9248.183944 PMC4924490 27398248

[6] Khan NU, Khan U, Khudadad U, Ali A, Raheem A, Waheed S, et al. Trends in mortality related to unintentional poisoning in the South Asian region from 1990 to 2019: analysis of data from the Global Burden of Disease Study. BMJ open. 2023;13(2):e062744. 36754559 10.1136/bmjopen-2022-062744 PMC9923325

[7] Gunnell D, Eddleston M, Phillips MR, Konradsen F. The global distribution of fatal pesticide self-poisoning: systematic review. BMC Public Health. 2007;7:1–15. 18154668 10.1186/1471-2458-7-357 PMC2262093

[8] Litchfield MH. Estimates of acute pesticide poisoning in agricultural workers in less developed countries. Toxicol Rev. 2005;24(4):271–8. 16499408 10.2165/00139709-200524040-00006

[9] Alwan IA, Brhaish AS, Awadh AI, Misnan A, Rahim NAA, Tangiisuran B, et al. Poisoning among children in Malaysia: A 10-years retrospective study. PLoS ONE. 2022;17(4):e0266767. 35482773 10.1371/journal.pone.0266767 PMC9049302

[10] Dai Q, Wang L, Gao X, Du D, Shuai P, Li L, Liu W. Clinical and epidemiological characteristics of acute poisoning in children in southwestern China: a review of 1755 cases from 2014 to 2020. Int J Gen Med. 2022:133–42. 10.2147/IJGM.S342253 PMC8749043 35027838

[11] Soave PM, Curatola A, Ferretti S, Raitano V, Conti G, Gatto A, et al. Acute poisoning in children admitted to pediatric emergency department: a five-years retrospective analysis. Acta Bio Medica: Atenei Parmensis. 2022;93(1):e2022004. 35315415 10.23750/abm.v93i1.11602 PMC8972869

[12] Khan NU, Pérez-Núñez R, Shamim N, Khan UR, Naseer N, Feroze A, et al. Intentional and unintentional poisoning in Pakistan: a pilot study using the Emergency Departments surveillance project. BMC Emerg Med. 2015;15:1–7. 26691609 10.1186/1471-227X-15-S2-S2 PMC4682408

[13] INTERNET. MINISTRY OF INFORMATION AND BROADCASTING (MOIB). PAKISTAN [Available from: https://moib.gov.pk/News/62983

[14] Ramesha K, Rao KB, Kumar GS. Pattern and outcome of acute poisoning cases in a tertiary care hospital in Karnataka, India. Indian J Crit care medicine: peer-reviewed official publication Indian Soc Crit Care Med. 2009;13(3):152. 10.4103/0972-5229.58541 PMC2823097 20040813

[15] Alva J, Devi ES, Chandrababu R, Abraham J, Balakrishnan JM. Epidemiologic characteristics, the length of hospital stay, and mortality of patients with organophosphate poisoning: a systematic review. Clin Epidemiol Glob Health. 2025;32:101932.

[16] Khonje V, Hart J, Venter J, Deonarain S, Grossberg S. Acute organophosphorus toxicity in a regional hospital in Johannesburg, South Africa: A retrospective chart review. Afr J Emerg Med. 2023;13(2):104–8. 37152660 10.1016/j.afjem.2023.04.002 PMC10160343

[17] Robb E, Baker M. Organophosphate Toxicity [Updated 2022 May 1]. StatPearls [Internet] StatPearls Publishing, Treasure Island, FL https://www ncbi nlm nih gov/books/NBK470430. 2023. 29261901

[18] Tarui T, Yoshikawa K, Miyakuni Y, Kaita Y, Tamada N, Matsuda T, et al. Independent risk factors for a complicated hospital course in intensive care unit overdose patients. Acute Med Surg. 2015;2(2):98–104. 29123701 10.1002/ams2.77 PMC5667207

[19] Aher AL, Shingade PU. Clinical Profile and in Hospital Outcome of Acute Poisoning Cases Admitted in Tertiary Care Hospital: A Prospective Observational Study. Vidarbha J Intern Med. 2023;33(1):3–9.

[20] Okazaki Y, Shimojo N, Matsuishi Y, Hoshino H, Ouchi A, Kawano S, et al. Risk factors for prolonged intensive care unit and hospital stay among patients with acute drug overdose in Japan. Acute Med Surg. 2020;7(1):e482. 31988794 10.1002/ams2.482 PMC6971431

[21] Shazia S, Jadoon OK, Zeb M, Abbasi S, Khan MA, ur Rashid H, et al. Medicolegal importance of organophosphorus poisoning in young adults. J Ayub Med Coll Abbottabad. 2024;36(2):346–9. 39609978 10.55519/JAMC-02-13257

[22] Amir A, Raza A, Qureshi T, et al. Organophosphate Poisoning: Demographics, Severity Scores and Outcomes From NationalPoisoning Control Centre, Karachi. Cureus. 2020;12(5):e8371. 10.7759/cureus.8371. 10.7759/cureus.8371 PMC7328692 32626615

[23] Shaikh S, Khaskheli MS, Meraj M, Raza H. Effect of Organophasphate poisoning among patients reporting at a tertiary healthcare facility of Sindh Pakistan. Pakistan J Med Sci. 2018;34(3):719. 10.12669/pjms.343.15000 PMC6041540 30034446

[24] Shah SH, Alia B, Muhammad K, Raza MA, Najeeb S. Clinical spectrum and outcome of children presenting with poisoning to tertiary care hospital. Pakistan J Physiol. 2021;17(4):15–8.

[25] Dabholkar S, Pirani S, Davis M, Khan M, Eddleston M. Suicides by pesticide ingestion in Pakistan and the impact of pesticide regulation. BMC Public Health. 2023;23(1):676. 37041526 10.1186/s12889-023-15505-1 PMC10088141

[26] WHO F. Preventing suicide: a resource for pesticide registrars and regulators. CMAJ. 2019;143:1–36.

[27] Organization WH. Suicide worldwide in 2021: global health estimates. World Health Organization; 2025.

[28] Perveen F, Ahmed N, Masud S, Ihsan MU, Khan UR, Khan NU. Parental knowledge attitude and practices about chemical and medicinal poisons: A hospital based study from Karachi, Pakistan. Injury. 2023;54:110481. 37573064 10.1016/j.injury.2022.11.024

[29] Zhang R, Cui N, Tang L, Pan Y, Li M, Yang G. A clinical review of acute poisoning among pediatric individuals. Eur J Med Res. 2026;31(1):29. 10.1186/s40001-025-03633-w PMC12777263 41331758

[30] Aljalahma N, Alabbasi L, Alkoheji H, Almahmood H, Aldoseri A, Husain F, et al. Accidental Ingestion of Intoxications (Drugs and Nondrug Materials) Among Pediatric Patients: A Multicenter Study in Bahrain Royal Medical Services Hospitals. J Toxicol. 2025;2025(1):2093893. 40963556 10.1155/jt/2093893 PMC12440636

[31] Abdel Baseer KA, Gad EF, Abdel Raheem YF. Clinical profile and outcome of acute organophosphate poisoning in children of Upper Egypt: a cross-sectional study. BMC Pediatr. 2021;21(1):98. 33637060 10.1186/s12887-021-02563-w PMC7908781

