# Supplementary figures and images for: Childhood and adolescent poisoning: a prospective analysis from the largest poison control centre
Source: BMC Pediatr. 2026 Jun 9;26:681. doi: 10.1186/s12887-026-07062-4 (PMC13393915; doi:10.1186/s12887-026-07062-4)

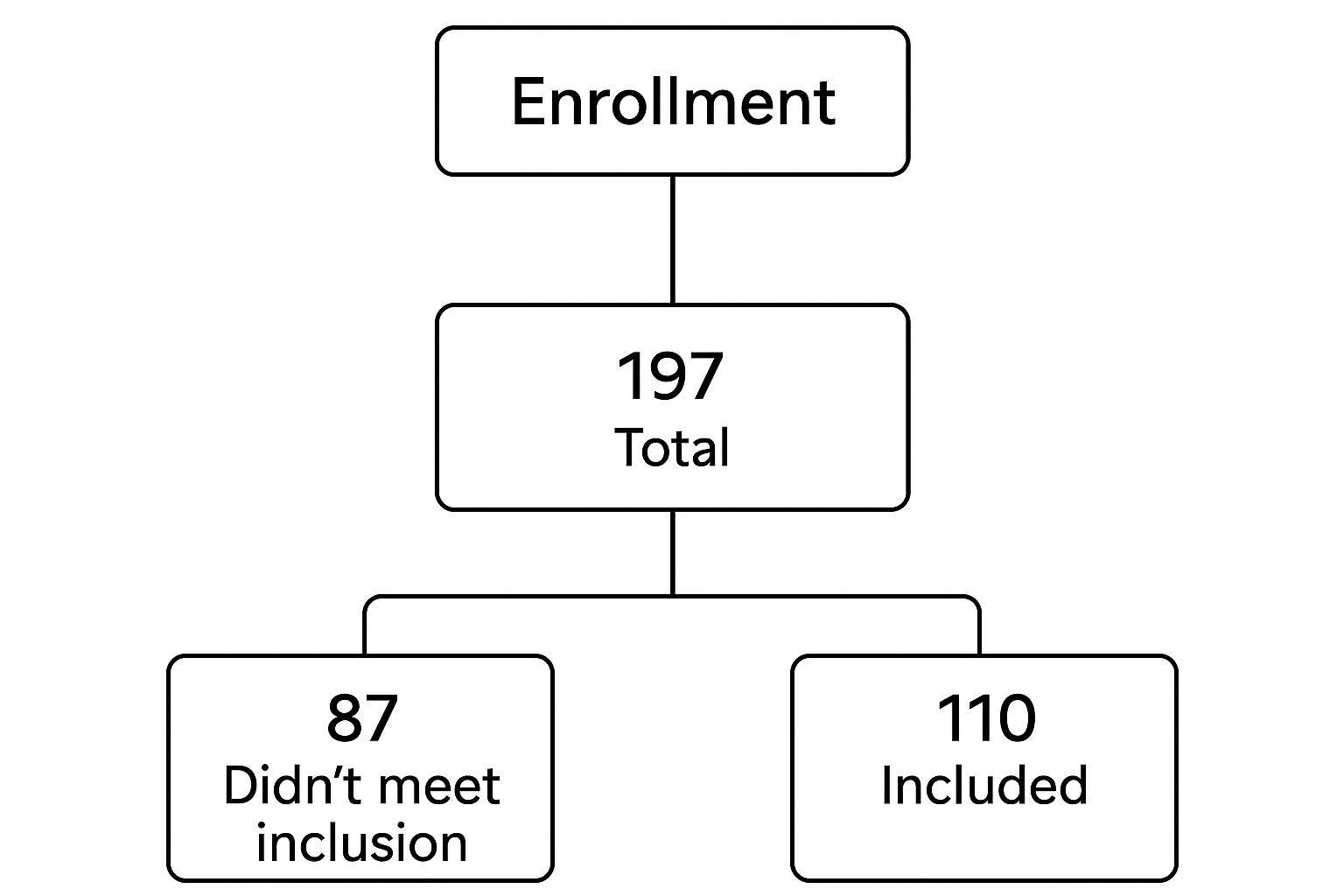

Supplement: Supplementary file 1 — Supplementary Material 1: Supplementary figure 1: Enrollment flow diagram. [file 12887_2026_7062_MOESM1_ESM.docx]
